# Do cognitive reserve proxies capture a common neural signature? A systematic review and meta-analysis of task-based fMRI studies

**DOI:** 10.1093/psyrad/kkag016

**Published:** 2026-04-17

**Authors:** Annachiara Crocetta, Jordi Manuello, Donato Liloia, Sergio Duca, Tommaso Costa, Franco Cauda

**Affiliations:** Functional Neuroimaging and Complex Neural Systems (FOCUS) Laboratory, Department of Psychology, University of Turin, Via Verdi 10, 10124 Turin, Italy; Translational Neuroimaging & Brain Connectivity Group, GCS-fMRI, Koelliker Hospital, C.so Galileo Ferraris 247, 10134 Turin, Italy; Neuroimaging & Data Science Group, GCS-fMRI, Koelliker Hospital, C.so Galileo Ferraris 247, 10134 Turin, Italy; Computational Neuroimaging & Complex Systems Group, GCS-fMRI, Koelliker Hospital, C.so Galileo Ferraris 247, 10134 Turin, Italy; Functional Neuroimaging and Complex Neural Systems (FOCUS) Laboratory, Department of Psychology, University of Turin, Via Verdi 10, 10124 Turin, Italy; Neuroimaging & Data Science Group, GCS-fMRI, Koelliker Hospital, C.so Galileo Ferraris 247, 10134 Turin, Italy; Department of Social and Human Science, University of Valle d’Aosta, Strada Cappuccini 2A, 11100 Aosta, Italy; Functional Neuroimaging and Complex Neural Systems (FOCUS) Laboratory, Department of Psychology, University of Turin, Via Verdi 10, 10124 Turin, Italy; Translational Neuroimaging & Brain Connectivity Group, GCS-fMRI, Koelliker Hospital, C.so Galileo Ferraris 247, 10134 Turin, Italy; Translational Neuroimaging & Brain Connectivity Group, GCS-fMRI, Koelliker Hospital, C.so Galileo Ferraris 247, 10134 Turin, Italy; Neuroimaging & Data Science Group, GCS-fMRI, Koelliker Hospital, C.so Galileo Ferraris 247, 10134 Turin, Italy; Computational Neuroimaging & Complex Systems Group, GCS-fMRI, Koelliker Hospital, C.so Galileo Ferraris 247, 10134 Turin, Italy; Functional Neuroimaging and Complex Neural Systems (FOCUS) Laboratory, Department of Psychology, University of Turin, Via Verdi 10, 10124 Turin, Italy; Computational Neuroimaging & Complex Systems Group, GCS-fMRI, Koelliker Hospital, C.so Galileo Ferraris 247, 10134 Turin, Italy; Neuroscience Institute of Turin (NIT), University of Turin, Via Verdi 10, 10124 Turin, Italy; Functional Neuroimaging and Complex Neural Systems (FOCUS) Laboratory, Department of Psychology, University of Turin, Via Verdi 10, 10124 Turin, Italy; Translational Neuroimaging & Brain Connectivity Group, GCS-fMRI, Koelliker Hospital, C.so Galileo Ferraris 247, 10134 Turin, Italy; Neuroimaging & Data Science Group, GCS-fMRI, Koelliker Hospital, C.so Galileo Ferraris 247, 10134 Turin, Italy; Computational Neuroimaging & Complex Systems Group, GCS-fMRI, Koelliker Hospital, C.so Galileo Ferraris 247, 10134 Turin, Italy; Neuroscience Institute of Turin (NIT), University of Turin, Via Verdi 10, 10124 Turin, Italy

**Keywords:** fMRI, cognitive reserve, brain aging, cognitive decline, PSI-SDM, neuroimaging

## Abstract

Cognitive reserve (CR) refers to the capacity of the brain to sustain cognitive performance despite age-related changes or pathophysiological conditions. Task-based functional magnetic resonance imaging (tb-fMRI) has been instrumental in exploring its neural correlates. CR is traditionally assessed through socio-behavioral proxies such as education, intelligence quotient, or composite indices. However, these proxies vary considerably in definition and application, posing challenges regarding their validity and consistency across experiments. This systematic review and meta-analysis examined whether these proxies are associated with consistent brain activation patterns in healthy adults. A literature search identified 12 eligible tb-fMRI experiments (*n* = 802 participants) reporting whole-brain CR-related activation. A coordinate-based meta-analysis using permutation of subject images—signed differential mapping (PSI-SDM) was conducted to assess consistent activation patterns across experiments, complemented by meta-regression analyses to examine whether differences in proxy type accounted for inter-study variability. The PSI-SDM meta-analysis yielded no significant clusters of activation. A qualitative synthesis of individual experiments further highlighted a lack of topographical consistency. These findings indicate that widely used socio-behavioral proxies are not associated with detectable, reproducible convergent patterns in tb-fMRI, revealing a measurement gap in current CR research. This null convergence likely reflects methodological heterogeneity and conceptual inconsistency in the literature, rather than the absence of CR-related neural mechanisms.

## Introduction

Humans are capable of a wide variety of behaviors and cognitive processes due to the dynamic nature of the brain. Successful cognition depends on the ability of the brain to adapt and reconfigure its networks in interaction with its environment. In the same way, a particular kind of brain reorganization makes it possible for the brain to recover and be resilient when it is damaged. This resilience is often attributed to the concept of “reserve,” and it is related to individual differences in the structural and functional mechanisms of the brain that influence cognitive and behavioral outcomes in response to aging and brain disease (Medaglia *et al*., 2017; Stern *et al*., 2020). While brain reserve is considered a passive capacity based on structural brain properties, cognitive reserve (CR) is an active process by which the brain reorganizes its networks (Alvares Pereira *et al*., 2022; Pappalettera *et al*., 2024) and employs compensatory mechanisms to maintain better-than-expected cognitive function despite age-related brain changes, injury, or disease (Barulli and Stern, 2013; Stern and Barulli, 2019; Boyle *et al*., 2023). Age-related neurophysiological changes include gray and white matter atrophy, vascular alterations, and functional disruptions in brain connectivity and neurotransmission. These changes affect cognitive functions, primarily including memory, processing speed, and attention (Harada *et al*., 2013; Toepper, 2017). However, individuals with higher CR demonstrate a greater ability to withstand these changes, delaying cognitive decline and reducing the clinical impact of neurodegenerative disease, such as Alzheimer’s disease (Cosentino and Stern, 2019; Kremen *et al*., 2022).

CR is a multifaceted construct arising from the interaction between genetic factors, such as variations in the BDNF gene and APOE allele, and modifiable environmental and lifestyle factors (Clare *et al*., 2017; Pettigrew and Soldan, 2019; Stern and Barulli, 2019; Pappalettera *et al*., 2024). This interplay unfolds dynamically across the lifespan through neuroplastic changes and compensatory mechanisms (Stern and Barulli, 2019), including neural efficiency, neural flexibility, and compensation. Neural efficiency refers to achieving comparable performance with lower recruitment of task-related networks, whereas neural flexibility captures the ability to modulate recruitment as task demand increases, including stronger engagement of the primary network and, when demand exceeds capacity, the involvement of additional resources. Compensation, in turn, refers to the advantageous engagement of alternative networks to sustain performance under challenge (Barulli and Stern, 2013; Ducharme-Laliberté *et al*., 2022). Importantly, these mechanisms are not fixed properties but dynamic and context-dependent processes that may vary across tasks, difficulty levels, and age-related neurocognitive constraints.

The assessment of CR is often done indirectly through proxies, which are practical measures that estimate an individual’s lifetime exposure to enriching cognitive experiences. These proxies provide measurable and practical ways for approximating CR, enabling researchers to investigate its impact on cognitive health and resilience (Tucker and Stern, 2014; Ko *et al*., 2020; Stern *et al*., 2020). Common proxies for CR include educational level, socioeconomic status, occupational complexity, and involvement in leisure and social activities (Nelson *et al*., 2021; Yang *et al*., 2022; Nijmeijer *et al*., 2023; Sanz Simon *et al*., 2023). Furthermore, intelligence quotient (IQ) and pre-morbid IQ, often measured using tools like the Wechsler Adult Intelligence Scale (WAIS-IV), are also used as proxies for CR, reflecting an individual’s baseline cognitive abilities and potential resilience to cognitive decline (Corral *et al*., 2006).

Although widely adopted, reliance on proxies presents methodological challenges (Howard *et al*., 2023). These measures often depend on self-reported data, which may introduce potential biases, and may not fully capture the dynamic and lifelong nature of CR. Furthermore, they may fail to reflect the underlying neurobiological mechanisms that contribute to cognitive resilience (Jones *et al*., 2011; Boyle *et al*., 2021). Although in 2019 the Collaboratory on Research Definitions for Reserve and Resilience in Cognitive Aging and Dementia was established to develop consensus definitions and research guidelines for this concept, measuring CR remains difficult due to the lack of standardized methods (Stern *et al*., 2020). Moreover, recent work by Kremen *et al*. (2022) emphasizes the need for congruity between conceptual and operational definitions, as inconsistencies arise when some proxies of CR (e.g. years or level of education or IQ) are used both as measures of reserve and as factors influencing cognitive performance. For example, using education as a proxy for CR may lead to confusion, as education itself does not inherently measure the adaptability of the brain but, rather, reflects life experiences that may contribute to CR (Kremen *et al*., 2022).

To address these limitations, researchers have attempted to combine socio-behavioral proxies with neuroimaging techniques, particularly functional magnetic resonance imaging (fMRI), to identify potential neural mechanisms underlying CR (Whalley *et al*., 2004; Franzmeier *et al*., 2017; Stern *et al*., 2018, 2021). This approach is based on the assumption that if CR reflects an individual’s ability to optimize neural resources and compensate for age-related or pathological changes, it should be reflected in distinct patterns of brain activation during cognitive tasks. By correlating CR proxies with fMRI activation, studies have sought to identify the neural substrates and functional mechanisms that support compensatory mechanisms and cognitive resilience. However, while tb-fMRI has been widely used in CR research (Anthony and Lin, 2018; Stern *et al*., 2021; Vockert *et al*., 2022; Boyle *et al*., 2023), its ability to capture the influence of CR on brain organization remains debated (Boyle *et al*., 2023; Mauti *et al*., 2024). CR is a lifelong, dynamic process, whereas fMRI studies are typically cross-sectional, capturing transient neural activity. This makes it uncertain whether observed activation patterns truly reflect the mechanisms underlying cognitive resilience rather than task-specific cognitive processes (Anthony and Lin, 2018; Mauti *et al*., 2024). Additionally, methodological variability across studies, including differences in the definitions of proxies, task paradigms, cognitive demands, and analytical techniques, complicates the identification of consistent neural correlates related to the construct of interest. Finally, the correlational nature of this approach does not clarify whether this combination of CR proxies and tb-fMRI activation can effectively capture CR or merely reflect individual differences in cognitive efficiency.

To systematically address these conceptual and methodological issues, the present study explicitly adopts an approach aimed at exploring heterogeneity in tb-fMRI studies of CR. Crucially, the objective is not to delineate the neural substrate of CR as a theoretical construct, but rather to evaluate whether the socio-behavioral proxies commonly used to operationalize CR (e.g. education, IQ, composite indices) yield convergent task-related activation patterns across studies.

A coordinate-based meta-analysis (CBMA) employing the permutation-subject images version of the signed differential mapping (PSI-SDM) method was conducted to assess the presence of consistent activation patterns linked to CR proxies across experiments, as well as targeted sensitivity and meta-regression analyses to further explore heterogeneity. Given the methodological heterogeneity in study design, cognitive tasks, and CR proxy definitions, a high degree of variability in results is expected. Thus, a failure to identify consistent neural correlates would not imply the absence of CR-related mechanisms per se; instead, it would underscore the limitations of current proxy-based approaches and highlight the need for revised conceptual models and more precise neuroimaging methodologies in CR research.

## Methods

The study design, encompassing the procedures for data selection and extraction, follows the established quality standards outlined in the PRISMA statement guidelines (Page *et al*., 2021) (Fig. S1, Fig. S2), and it aligns with the current best-practice rules for CBMA (Müller *et al*., 2018; Manuello *et al*., 2022a).

### Literature search strategy

A systematic literature search was conducted across three major databases (PubMed, Google Scholar, and Scopus) up until February 2025. This strategy aimed to encompass the wide range of existing studies on CR and its relationship with fMRI. The search terms employed were specifically tailored to each database to ensure thorough coverage. For PubMed, the query utilized was: (“cognitive reserve” [Title/Abstract] AND fMRI) NOT review [Publication Type]. In Google Scholar, the search was framed as “cognitive reserve” AND “fMRI task,” while for Scopus, the terms were “cognitive reserve” AND “fMRI.” In addition to database searches, the reference lists of the included studies were reviewed to identify any further relevant publications that may have been missed.

### Eligibility criteria, study selection, and data extraction

Peer-reviewed studies that focused on healthy adult participants (i.e. age > 18 years) undergoing tb-fMRI were included. The focus on healthy individuals was chosen to exclude populations with cognitive impairments or neurological conditions, allowing for a more precise examination of how CR influences brain function in a normative context. CR was operationalized through various proxies, such as education, occupational complexity, leisure activities, and IQ. Inclusion criteria required studies to report at least one quantitative measure of CR alongside fMRI as the imaging modality. Only peer-reviewed articles published in English were included. Studies had to report brain activation using whole-brain analyses and present results in standard stereotactic space (Talairach or MNI). Exclusion criteria encompassed single-subject reports, region of interest (ROI) analyses, structural imaging studies, or those involving pathological populations (Müller *et al*., 2018; Manuello *et al*., 2022a).

It is important to note that in the context of CBMA, the term “experiment” refers to a single analysis of imaging data that evaluates a specific contrast, identifying areas of brain activity related to a particular task or condition (Laird *et al*., 2011; Eickhoff *et al*., 2012). A “study” may include one or more such experiments. Studies like those by Springer *et al*. (2005) and Rodríguez-Aranda *et al*. (2020) examined different participant groups performing the same cognitive tasks. Since in these cases results from each group were reported as independent contrasts, two distinct experiments per study were included in the current meta-analysis. The analysis employed within-group contrasts to investigate how different levels of CR proxies affected brain activation during task performance. Specifically, two types of contrasts were considered. The first was a positive correlation, where higher CR levels were associated with increased task-related brain activation. The second contrast examined a negative correlation, where higher CR levels corresponded to lower task-related brain activation.

Two authors (C.A. and M.J.) carried out the initial selection of articles in accordance with predetermined criteria and subsequently assessed independently the full-text eligibility. Discrepancies were resolved through discussion under the supervision of the senior author (C.F.).

### Data analysis

The primary analysis of the current study consists of a CBMA through the PSI-SDM method. Additionally, a range of meta-regressions and CBMAs on sub-samples were performed as complementary analyses.

#### CBMA with the PSI-SDM algorithm

To assess potential consistent activation patterns associated with CR proxies across experiments, a CBMA was conducted using the PSI-SDM algorithm (version 6.23). This method reconstructs voxel-wise effect size maps from the reported peak coordinates and associated statistical values (e.g. *t*-values, *z*-scores, or *P*-values) from individual experiments, applying an anisotropic Gaussian kernel with a fixed full-width at half maximum of 10 mm (Albajes-Eizagirre *et al*., 2019). The final meta-analytic map is generated through a random-effects model and corrected for multiple comparisons using family-wise error (FWE) with 1000 permutations and threshold-free cluster enhancement (TFCE) statistics (*P* ≤ 0.05; minimum cluster size = 10 voxels), in line with methodological recommendations for voxel-based neuroimaging meta-analyses (Smith and Nichols, 2009; Albajes-Eizagirre *et al*., 2019). Unlike other CBMA approaches, such as activation likelihood estimation (Eickhoff *et al*., 2016; Müller *et al*., 2018), PSI-SDM has been effectively applied to datasets with a relatively small number of experiments, showing negligible bias and stable estimation of activation patterns (Albajes-Eizagirre *et al*., 2019; Liloia *et al*., 2022; Sun *et al*., 2023; Liloia, Manuello, *et al*., 2024; Liloia, Zamfira, *et al*., 2024). Further information on the PSI-SDM toolbox and procedures is available at www.sdmproject.com/sdmtools/.

#### Reliability and heterogeneity analyses

To assess the reliability of identified alteration clusters, a voxel-wise Jack-knife analysis was conducted. This approach involved iteratively removing one experiment at a time from the dataset to determine the persistence of significant or null results and evaluate the impact of each experiment on the overall findings. The reliability of a finding was established when it remained significant across the majority or all combinations of experiments (Radua and Mataix-Cols, 2009).

Moreover, a further inter-studies heterogeneity analysis was designed based on the computation of I^2^ statistic within a random-effects model, where an *I*^2^ value below 50% indicates low heterogeneity (Egger *et al*., 1997). To this aim, CBMA-derived peak coordinate value has to be extracted for each resulting statistically significant cluster

Although PSI-SDM was designed to jointly model experiments reporting results with opposite sign, two secondary CBMAs were conducted, separating experiments reporting positive associations (where higher CR levels are associated with increased task-related brain activation) from those reporting negative associations (where higher CR levels corresponded to lower task-related brain activation), to rule out any possible cancellation effect between different mechanisms.

To partially mitigate task-driven heterogeneity within proxy categories, a domain-restricted sensitivity analysis was additionally performed focusing on memory paradigms (working, episodic, and semantic memory). Experiments were included if the task demands primarily targeted memory processes, thereby increasing comparability in terms of expected task-relevant networks and cognitive requirements. The PSI-SDM pipeline and statistical inference followed the same procedures adopted for the main analysis, including TFCE-based correction for multiple comparisons.

As a further way to test heterogeneity, hierarchical clustering was applied to the maps representing the experiments (Manuello et al., 2022b,c). However, in light of their limited number (*N* = 12), results were considered exploratory, and were therefore described in the Supplementary Materials (together with more methodological details).

#### Meta-regression analysis with PSI-SDM

To further examine the possible influence of different CR proxies on brain activation patterns, an additional meta-regression analysis using PSI-SDM was conducted. This approach assesses voxel-wise relationships between activation patterns and experiment-level variables, providing insight into how CR proxies modulate neural activity (Radua *et al*., 2012). The meta-regression was performed separately for IQ and education variables that were extracted from the demographic and methodological details reported in the included experiments. By contrast, it was not possible to conduct a meta-regression for experiments using the composite score due to the lack of a standardized numerical value across experiments.

To address concerns about age-related heterogeneity, an additional meta-regression was conducted using experiment-level mean age as a continuous moderator. This analysis was intended to test whether the magnitude and spatial distribution of proxy-related task activation varied systematically as a function of age, without imposing an arbitrary age stratification.

All meta-regression results were thresholded at an uncorrected *P*-value of ≤ 0.0005 with a minimum cluster size of 10 voxels, in accordance with the recommendations of the SDM team to optimize the balance between specificity and sensitivity (Radua *et al*., 2012). These additional analyses provide complementary insights beyond the primary meta-analytic framework, elucidating the role of study-level variables in modulating observed activation patterns.

## Results

The systematic literature search identified 1657 articles. After removal of duplicates and screening of titles and abstracts, 24 articles were assessed for eligibility at the full-text level. Of these, 14 were excluded based on the predetermined inclusion and exclusion criteria, leaving 10 studies that met eligibility criteria (Stern *et al*., 2018, 2003; Springer *et al*., 2005; Waiter *et al*., 2008; Bartrés-Faz *et al*., 2009; Solé-Padullés *et al*., 2009; Archer *et al*., 2018; Elshiekh *et al*., 2020; Rodríguez-Aranda *et al*., 2020; Ducharme-Laliberté *et al*., 2022). These studies reported a total of 12 independent tb-fMRI experiments, which included 802 healthy participants and reported 96 peak activation coordinates.

Demographic characteristics of the included participants are summarized in Table 1, and details on study samples, experimental tasks, and primary findings are provided in Table 2 (more details about activation foci coordinates and *t*-values extracted from each included study are provided in Table S1). The PRISMA flow diagram is presented in Fig. S1, and the PRISMA 2020 checklist is available in Fig. S2.

**Table 1:** Experiments included in the systematic review: sample size demographic data for each tb-fMRI study.

Author, Year	Sample size (*N*)	% Female	Mean age (YRS)	Age range (YRS)
Archer *et al.*, (2018)	189	N/A	N/A	21–79
Bartrés-Faz *et al.*, (2009)	15	73	68.3	N/A
Ducharme-Laliberté *et al.*, (2022)	39	58	73.11	65–88
Elshiekh *et al.*, (2020)	154	71	48.08	19–76
Rodríguez-Aranda *et al.*, (2020)*young	15	47	26.8	22–36
Rodríguez-Aranda *et al.*, (2020)*old	27	67	70.6	65–76
Solé-Padullés *et al.*, (2009)	16	55	73.31	>65
Springer *et al.*, (2005)*young	14	50	23.4	18–30
Springer *et al.*, (2005)*old	19	53	73.9	>65
Stern *et al.*, (2003)	19	N/A	N/A	18–30
Stern *et al.*, (2018)	255	54	N/A	20–80
Waiter *et al.*, (2008)	40	N/A	68	N/A

**Table 2:** Characteristics of studies included in the review and meta-analyses, detailing sample size, number of activation foci, CR proxies, fMRI tasks, and main findings.

Author, Year	Sample size	Foci	CR proxies	fMRI task domain	fMRI task name/paradigm	Key findings
Archer *et al.*,(2018)	189	4	Education	Working memory	Spatial addition task (SAT)	Increased activations in the L superior temporal gyrus, R cuneus and L cerebellum
Bartres-Faz *et al.*,(2009)	15	1	Composite score	Working memory	N-back paradigm	Decreased activations in the R inferior frontal cortex
Ducharme-Laliberté *et al.*,(2022)	39	2	Education	Working memory	N-back paradigm	Increased activations in the R caudate nucleus.Decreased activation in the L medial superior frontal gyrus
Elshiekh *et al.*,(2020)	154	3	Composite score	Episodic memory	Context memory task	Increased activations in the L superior temporal gyrus and R cuneus.Decreased activation in the L dorsolateral prefrontal cortex
Rodríguez-Aranda *et al.*,(2020)*young	15	1	Composite score	Language/Semantic memory	Silent semantic verbal fluency task	Decreased activations in the R hemisphere in the visual cortex, cingulum, optic radiation, and callosal body
Rodríguez-Aranda *et al.*,(2020)*old	27	4	Composite score	Language/Semantic memory	Silent semantic verbal fluency task	Increased activations of frontal regions (LR middle frontal gyrus and superior frontal gyrus), L inferior parietal lobule and LR superior parietal lobule, LR precuneus cortex and L cingulate gyrus
Sole-padulles *et al.*,(2009)	16	10	Composite score	Episodic memory	Visual encoding task	Decreased activations in the LR frontal lobe and the cerebellum, R temporal cortex and the L thalamus
Springer *et al.,*(2005)*young	14	17	Education	Episodic memory	Encoding—recognition memory paradigms	Increased activations in the activity in mostly posterior areas such as the LR posterior cingulate gyrus, cuneus and precuneus, and lateral and medial temporal regions
Springer *et al.*,(2005)*old	19	7	Education	Episodic memory	Encoding—recognition memory paradigms	Increased activations in the R areas of the temporal and parietal cortices and cingulate gyrus, LF prefrontal areas
Stern *et al.*,(2003)	19	36	IQ	Episodic memory	Nonverbal recognition task	Increased activations in the L middle frontal gyrus, L postcentral gyrus, and R medial frontal gyrus.Decreased activations in the LR medial frontal gyrus, superior frontal gyrus, postcentral gyrus, and precentral gyrus; R insula, inferior parietal lobule, and claustrum; L middle temporal gyrus, superior temporal gyrus, thalamus, superior parietal lobule, and parahippocampal gyrus.
Stern *et al.*,(2018)	255	8	IQ	Vocabulary,Perceptual Speed,Fluid Reasoning,Episodic Memory	Synonyms task,Antonyms task,Picture Naming task,Digit Symbol task,Letter Comparison task,Pattern Comparison task,Paper Folding task,Matrix Reasoning task,Letter Sets task,Logical Memory task,Word Order Recognition task,Paired Associates task	Increased activations in the LR cerebellum, LR medial frontal gyrus, LR ACC, and LR superior temporal gyrus.Decreased activations in the LR inferior parietal lobule, LR inferior frontal gyrus, and L precuneus
Waiter *et al.*,(2008)	40	3	IQ	Processing speed	Inspection Time task	Increased activation in the R ACC

Abbreviations: ACC, anterior cingulate cortex; IQ, intelligence quotient.

### Findings from the literature

The following section provides a concise overview of the main findings from the literature regarding the included studies. The results are organized by the proxies used to assess CR, which can be categorized into three groups: education, IQ, and composite scores incorporating multiple indices.

Education was uniformly operationalized as years of formal education, a definition consistent with standard practice in CR research and widely adopted in neuropsychology (e.g. Farfel *et al*., 2013; Bruno *et al*., 2014; Kochhann *et al*., 2023). Although this measure does not capture qualitative aspects of education, such as degree type or educational quality, its use provides a reliable and comparable index of lifelong cognitive enrichment across diverse samples. For this reason, years of education remain the most frequently employed and validated proxy in the field, offering a pragmatic basis for cross-study comparisons. Experiments using education as a proxy (Springer *et al*., 2005; Archer *et al*., 2018; Ducharme-Laliberté *et al*., 2022) demonstrated both greater and lesser activation within the frontal cortex, particularly in older adults. These changes were observed in regions such as the left medial superior frontal gyrus and bilateral prefrontal areas (Springer *et al*., 2005; Ducharme-Laliberté *et al*., 2022). Greater activation was also found in temporal regions, including the left superior and bilateral medial temporal areas (Springer *et al*., 2005; Archer *et al*., 2018). For example, Archer *et al*. (2018) found that individuals with higher education showed enhanced accuracy and reduced response times in a spatial working memory task, which was accompanied by increased neural engagement in regions such as the temporal gyrus, cuneus, and cerebellum. Similarly, Ducharme-Laliberté *et al*. (2022) observed greater activation in the right caudate nucleus, while (Springer *et al*., 2005) highlighted age-related differences in the correlation between education and brain activation during a verbal episodic memory task. Younger individuals with higher education exhibited lesser frontal activation but greater medial temporal activation, whereas older adults showed greater frontal activation.

IQ was primarily estimated using standardized instruments such as the Wechsler Adult Intelligence Scale (WAIS), reflecting baseline cognitive efficiency and problem-solving ability. Studies adopting IQ as a CR proxy (Stern *et al*., 2003, 2018; Waiter *et al*., 2008) highlighted significant involvement of the frontal cortex, particularly the medial and inferior frontal gyri bilaterally. For example, Stern *et al*. (2003) found reduced activation in frontal regions during a nonverbal recognition test among individuals with higher IQ, suggesting greater neural efficiency during task performance. Furthermore, these studies reported variable activation patterns depending on task demands, with the anterior cingulate cortex (ACC) often implicated in efficient cognitive control. Lesser activation in the parietal cortex, particularly the inferior and superior parietal lobules, was also noted across studies (Stern *et al*., 2018).

Composite scores were employed in several studies to overcome the limitations of single proxies and to provide a more comprehensive assessment of CR by capturing multiple facets of lifelong enrichment. However, their operationalization varied considerably: Rodríguez-Aranda *et al*. (2020) combined education with leisure and cognitive activities; Ducharme-Laliberté *et al*. (2022) adopted the Cognitive Reserve Index, which integrates education, occupational complexity, and leisure engagement; Elshiekh *et al*. (2020) derived a composite index including education, occupation, and social/leisure activities; and Solé-Padullés *et al*. (2009) constructed a score combining education, estimated IQ, and socio-cultural variables. Even with varying definitions of composite proxies, these studies showed the frontal cortex again as a central ROI, with predominantly lesser activations. These findings suggest greater neural efficiency in bilateral frontal regions among individuals with higher CR. For example, Bartrés-Faz *et al*. (2009) identified a negative correlation between CR and fMRI signal during a working memory task in the right inferior frontal cortex. Similarly, lesser cerebellar activation was observed in the study by Solé-Padullés *et al*. (2009), indicating that memory demands could modulate activity in memory-related networks.

#### fMRI task paradigms of studies included

The included fMRI studies covered a set of recurring task paradigms. Working memory was mainly assessed with N-back paradigms (Bartrés-Faz *et al*., 2009; Ducharme-Laliberté *et al*., 2022), which require continuous monitoring and updating of stimuli across different load conditions. One study used a Spatial Addition Task (Archer *et al*., 2018), a visuospatial working-memory paradigm requiring maintenance and manipulation of spatial information. Episodic memory tasks largely followed encoding/recognition designs, including a visual encoding paradigm (Solé-Padullés *et al*., 2009) and a context-memory task involving item–context binding during encoding and subsequent retrieval (Elshiekh *et al*., 2020). In addition, Springer *et al*. (2005) used encoding and recognition memory paradigms to assess episodic memory across age groups, whereas Stern *et al*. (2003) employed a nonverbal recognition task. Language/semantic processing was probed with a silent (covert) semantic verbal fluency task, implemented in a block design interleaving semantic generation with finger tapping and rest (Rodríguez-Aranda *et al*., 2020). Processing speed was assessed with an inspection time task requiring a simple visual discrimination under brief stimulus exposure followed by backward masking (Waiter *et al*., 2008). Finally, in the multi-domain battery study (Stern *et al*., 2018), additional tasks targeted vocabulary, perceptual speed, fluid reasoning, and episodic memory through standard paradigms (e.g. synonyms/antonyms judgments, picture naming, digit symbol and comparison tasks, matrix reasoning, and episodic memory tests).

### Quantitative analyses

#### PSI-SDM meta-analysis

PSI-SDM analysis did not reveal any significant clusters when applying the TFCE at *P* ≤ 0.05 with a minimum cluster size of 10 voxels.

#### Reliability and heterogeneity analyses

Voxel-wise jack-knife sensitivity analysis confirmed that no clusters emerged in any leave-one-out iteration analyses (Table S2), thereby excluding a scenario in which results were driven by single influential experiments.

Furthermore, given that the CBMA produced no significant clusters, the heterogeneity assessment could not be conducted as planned, because *I*² is defined relative to statistically significant meta-analytic peaks. Consequently, no interpretable estimates of between-study heterogeneity were obtained.

In stratified PSI-SDM analyses, no clusters survived TFCE-FWE correction in either the positive-association subgroup (association between higher CR proxy level and higher activation; 9 experiments) or the negative-association subgroup (association between higher CR proxy level and lower activation; 6 experiments). These results suggest that the absence of voxel-wise convergence in the primary CBMA is unlikely to be explained solely by pooling experiments with opposite directions of association, possibly associated with different neurocognitive mechanisms.

In the domain-restricted sensitivity analysis limited to memory paradigms, no clusters survived TFCE-based correction for multiple comparisons.

#### Meta-regression analysis with PSI-SDM

The meta-regression analyses conducted with PSI-SDM to examine the relationship between voxel-wise activation patterns and the proxies of CR did not yield statistically significant results. Specifically, no clusters survived the predefined statistical threshold at an uncorrected *P*-value of <0.0005 with a minimum cluster size of 10 voxels for either education or IQ.

The additional meta-regression including mean age as an experiment-level moderator did not yield any clusters surviving the predefined statistical threshold at an uncorrected *P*-value of <0.0005 with a minimum cluster size of 10 voxels.

## Discussion

Despite the growing reliance on tb-fMRI paradigms to assess the neural substrates of CR (Colangeli *et al*., 2016; Anthony and Lin, 2018; Stern *et al*., 2021; Boyle *et al*., 2023; Mauti *et al*., 2024), this meta-analytic investigation did not identify consistent and reproducible patterns of brain activation across experiments. These findings may be interpreted in two complementary ways. On the one hand, they are consistent with prior evidence suggesting that CR may not represent a unitary construct, but rather a dynamic interplay of multiple factors, which cannot be fully captured by isolated socio-behavioral proxies (Marques *et al*., 2016; van Loenhoud *et al*., 2017; Lee *et al*., 2019; Hasanzadeh *et al*., 2025). On the other hand, the present null results are expected and mainly reflect the intrinsic heterogeneity of the available literature, including variability in proxy definitions, task paradigms, analytic strategies, and sample size and characteristics. This heterogeneity complicates the identification of reproducible neural correlates and suggests that the absence of consistent clusters should not be interpreted as definitive evidence against the existence of neural mechanisms supporting CR, but rather as an indication of a measurement gap, whereby current proxy-based tb-fMRI approaches may be too coarse to detect CR-related neural processes.

The PSI-SDM null findings were corroborated by the machine learning results. The hierarchical clustering revealed high overall dissimilarity among activation patterns, with a non-coherence score of 0.994, indicating minimal voxel-wise similarity between experiments. Interestingly, two experiments, the negative contrasts reported by Bartrés-Faz *et al*. (2009) and Rodríguez-Aranda *et al*. (2020) showed relatively higher similarity. This may suggest that composite CR proxies, by capturing a broader and less specific range of cognitive processes, may facilitate the detection of commonalities across studies. The absence of strong clustering based on task type or proxy further suggests that the observed patterns are not primarily driven by these categorical variables, but rather by a more complex interplay of methodological and cognitive factors.

**Figure 1:**
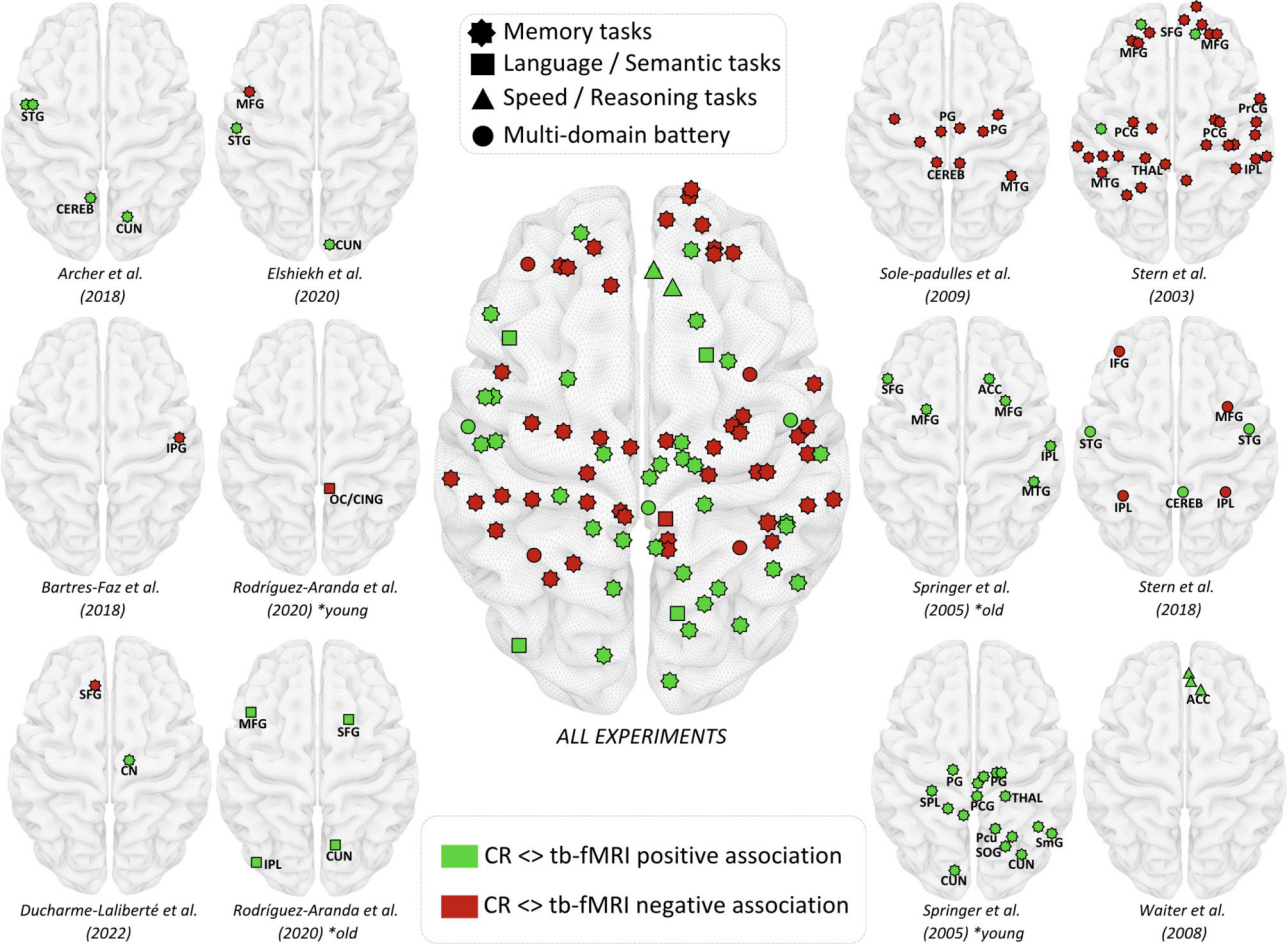
Visualizations of main brain activations found in each experiment. Abbreviations: ACC, anterior cingulate cortex; STG, superior temporal gyrus; CEREB, cerebellum; CUN, cuneus; IPG, inferior precentral gyrus; CN, caudate nucleus; SFG, superior frontal gyrus; MFG, middle frontal gyrus; OC, occipital cortex; CING, cingulum; IPL, inferior parietal lobule; MTG, middle temporal gyrus; THAL, thalamus; PG, precentral gyrus; PCG, posterior cingulate gyrus; SOG, superior occipital gyrus; SmG, supramarginal gyrus; Pcu, precuneus; SPL, superior parietal lobule; PrCG, precentral gyrus; IFG, inferior frontal gyrus.

Although the quantitative meta-analytic results did not reveal any detectable, reproducible convergence associated with CR proxies, the qualitative review of individual experiments allows for cautious, hypothesis-generating considerations. Several experiments identified modulations in prefrontal regions, often interpreted as reflecting neural efficiency or compensatory mechanisms (Ansado *et al*., 2012; Vermeij *et al*., 2014; Hakun *et al*., 2015; Holtzer *et al*., 2022). For instance, some studies reported lower frontal activation in individuals with higher CR, potentially indicating reduced resource demands during task performance (Bartrés-Faz *et al*., 2009; Ducharme-Laliberté *et al*., 2022). Others, however, described increased activation in frontal or medial temporal areas (sometimes within the same study) highlighting the task-dependent and context-sensitive nature of these effects. Overall, the activations from the original experiments showed the lack of topographical consistency: activation differences were reported in cortical and subcortical areas across frontal, temporal, parietal, and cerebellar regions, with both positive and negative contrasts depending on the study (see Fig. 1).

### Open issues in the study of CR

The current neuroimaging literature on CR presents several issues. One of these is the limited number of available tb-fMRI studies and experiments investigating CR proxies in healthy individuals. Small sample sizes, both within and across studies, reduce the capacity to account for inter-individual variability, a key concern in neuroimaging research, where statistical power is critical for reliable detection of activation patterns (Manuello *et al*., 2022a).

A second challenge concerns the heterogeneity in how CR is operationalized. The use of different proxies and the variability in tb-fMRI designs make it difficult to draw definitive conclusions about the neural mechanisms underlying CR. For example, lifestyle proxies may not directly measure brain adaptability but instead serve as indirect markers of the enriching experiences that shape CR over a lifetime. This disconnection between the conceptualization of CR and its operationalization through proxies raises fundamental questions about the validity of these measures in capturing the true essence of cognitive resilience. While it is established that certain lifestyle proxies, such as education, influence cognitive performance and decline (Hall *et al*., 2007; Hindle *et al*., 2014; Clare *et al*., 2017; Ko *et al*., 2020; Manuello *et al*., 2024), understanding the underlying neural mechanisms of cognitive resilience during physiological aging is more complex. The assumption that a higher level of education equates to a higher level of neural efficiency or flexibility overlooks the complex interplay of genetic, environmental, and neurobiological factors that contribute to CR. Consequently, relying solely on socio-behavioral proxies risks oversimplifying the nuanced processes of neural compensation and cognitive maintenance, which are likely more deeply rooted in brain network dynamics than in static measures of educational attainment (Marques *et al*., 2016; van Loenhoud *et al*., 2017; Lee *et al*., 2019; Hasanzadeh *et al*., 2025).

This heterogeneity within proxy categories may further contribute to the null meta-regression findings. Even when the same proxy is used (e.g. education or IQ), experiments differ substantially in paradigm structure, contrast definitions, and cognitive demands; as a result, proxy-related modulation may be expressed within task-relevant systems but may not generalize topographically across divergent tasks. This proxy-by-task interaction can attenuate coordinate-level convergence and reduce the sensitivity of experiment-level meta-regression, which necessarily collapses across rich within-paradigm variability. In line with this interpretation, a domain-restricted sensitivity analysis focusing on memory paradigms was conducted to increase comparability of cognitive demands; however, no effects survived TFCE correction.

Tb-fMRI designs add an additional layer of complexity. Different cognitive tasks activate distinct neural networks, and the same CR proxy may manifest differently depending on the cognitive demands of the task at hand. This makes it problematic to draw definitive conclusions about the neural mechanisms of CR, as a single task-based paradigm may not capture the full spectrum of cognitive processes that CR influences. A proxy like education may correlate with greater neural efficiency in one task but show no such effect in another, which underscores the limitations of relying on one-size-fits-all measures for such a multifaceted construct.

Additionally, the age range of participants included in these studies is often broad and heterogeneous, which may obscure age-specific effects of CR by increasing between-experiment variability and averaging across potentially distinct neurocognitive stages. It is likely that CR operates differently across the lifespan (Wang *et al*., 2017; Pettigrew and Soldan, 2019), with reserve-related modulation becoming more salient under greater neural challenge and with age-related changes in network recruitment and efficiency. In this context, pooling young and older adults within the same evidence base may attenuate convergence and mask proxy-by-age (and potentially proxy-by-task) interactions. Future research should therefore prioritize more narrowly defined age bands, harmonized task paradigms, and more complete reporting of age distributions to better characterize when and how CR shapes task-evoked activation.

### Methodological considerations

Coordinate-based meta-analytic (CBMA) techniques have an inherent limited resolution, as they focus on reporting significant coordinates rather than analyzing the entire voxel-wise statistic parametric maps, due to the limited public availability in published studies (Manuello *et al*., 2022a). Nonetheless, this approach remains well-established and widely used in the field (Eickhoff *et al*., 2009; Radua *et al*., 2012). Not less important, the cross-sectional nature of most studies restricts the ability to explore how brain activation patterns related to CR evolve over time. Future longitudinal studies, which scan the same participants at multiple points across their lifespan, could offer a more nuanced understanding of how CR develops and changes with age.

In addition, the relatively small number of eligible experiments limits statistical power and the precision of random-effects estimation, increasing the likelihood of Type II error and reducing sensitivity to subtle moderation effects. It should also be noted that, although a CBMA framework was applied, the present work differed from canonical CBMAs in its conceptual focus. Traditional CBMAs usually aggregate homogeneous tb-fMRI experiments investigating the same construct, under the assumption that conceptual and methodological coherence enhances statistical convergence. In contrast, the current study intentionally reflected the actual heterogeneity of the CR literature by including all tb-fMRI experiments that explicitly operationalized CR through socio-behavioral proxies. The methodological rationale was therefore not to enforce homogeneity, but rather to empirically test whether these studies, despite their diversity in proxy definitions and task paradigms, showed any degree of neural coherence.

Moreover, the evaluation of age-related moderation was constrained by reliance on experiment-level summary indices (i.e. mean age), which do not capture within-sample age dispersion or potential nonlinear lifespan effects, and may therefore reduce sensitivity to detect age-dependent modulation. Similarly, composite CR proxies were operationalized heterogeneously across studies (e.g. education combined with vocabulary, IQ, or other indicators), likely introducing additional measurement noise and attenuating the detectability of moderation effects. Finally, although the absence of preregistration could be seen as a limitation, the primary analyses and sensitivity tests were conducted transparently and in line with established CBMA recommendations (Müller *et al*., 2018; Manuello *et al*., 2022a). Moreover, the complete set of foci analyzed is reported in supplementary Table S1.

### Toward a network-based approach in CR research

The inconsistencies emerging from proxy-based tb-fMRI studies suggest that CR cannot be adequately understood through isolated measures or task-specific activations. A shift toward a network-based perspective may provide a more comprehensive account of the mechanisms supporting cognitive resilience. Network neuroscience conceptualizes the brain as a system of interconnected regions, where properties such as efficiency, modularity, and flexibility can be quantified (Rubinov and Sporns, 2010; Steffener and Stern, 2012; Benson *et al*., 2018). Evidence from both task-based and resting-state studies indicates that individuals with higher CR often exhibit more efficient and adaptable network configurations, particularly in large-scale systems such as the default mode and fronto-parietal networks (Schultz and Cole, 2016; Franzmeier *et al*., 2017; Hartwigsen, 2018; Stern *et al*., 2018). Within this perspective, resting-state connectivity may capture the intrinsic organizational features that enable resilience, while tb-fMRI reflects their task-specific manifestations. Together, these approaches suggest that neural mechanisms underlying CR are not static properties but emerge from the dynamic interplay of large-scale networks changes (Hillary and Grafman, 2017; Lee *et al*., 2019). Future research combining network-level analyses across modalities will be essential to clarify how CR supports cognitive function, how it adapts with aging, and how it may buffer against disease-related decline.

## Conclusion

This systematic review and meta-analysis examined whether commonly used proxy measures of CR, such as education, IQ, and composite indices, show convergent task-related activation patterns in healthy individuals. Despite growing interest in investigating CR through the association between socio-behavioral proxies and task-based fMRI, the multi-method analyses conducted here did not identify statistically significant or topographically consistent convergence across experiments. The lack of detectable, reproducible convergence in activation patterns suggests a potential measurement gap: traditional socio-behavioral proxies, while valuable at the behavioral level, may lack the precision needed to capture the neural-level adaptability that CR is theorized to reflect. This study shows the current empirical limits of proxy-based fMRI research on CR, offering a foundation for its conceptual refinement. Progress in this field will require more standardized operationalization of CR, greater methodological consistency, and the incorporation of network-based approaches capable of probing the distributed and dynamic nature of CR. By addressing these challenges, future research may more effectively delineate the neural mechanisms that support CR and clarify their role in sustaining cognitive health across the lifespan.

## Supplementary Material

kkag016_Supplemental_File
