# Supplementary material for: Do cognitive reserve proxies capture a common neural signature? A systematic review and meta-analysis of task-based fMRI studies
Source: Psychoradiology. 2026 Apr 17;6:kkag016. doi: 10.1093/psyrad/kkag016 (PMC13244274; doi:10.1093/psyrad/kkag016)
Supplement: kkag016_Supplemental_File [file kkag016_supplemental_file.docx]

#### **Do Cognitive Reserve Proxies capture a common neural signature? A Systematic Review and Meta-Analysis of Task-Based fMRI Studies**

Annachiara Crocetta^1,2,3,4^, Jordi Manuello^1,3,6*^, Donato Liloia^1,2^, Sergio Duca^2,3,4^,

Tommaso Costa^1,4,5^, Franco Cauda^1,2,3,4,5^

^1^Functional Neuroimaging and Complex Neural Systems (FOCUS) Laboratory, Department of Psychology, University of Turin, Turin, Italy

^2^Translational Neuroimaging & Brain Connectivity Group, GCS-fMRI, Koelliker Hospital, Turin, Italy

^3^Neuroimaging & Data Science Group, GCS-fMRI, Koelliker Hospital, Turin, Italy

^4^Computational Neuroimaging & Complex Systems Group, GCS-fMRI, Koelliker Hospital, Turin, Italy

^5^Neuroscience Institute of Turin (NIT), Turin, Italy

^6^Department of Social and Human Science, University of Valle D’Aosta, Aosta, Italy

Corresponding author:

Jordi Manuello, PhD
FocusLab and GCS fMRI, Koelliker Hospital and Department of Psychology
University of Turin
E-mail: [jordi.manuello@unito.it](mailto:jordi.manuello@unito.it)

**Supplementary Materials**

**Methods s1** Creation of experiment level maps for Unsupervised Machine Learning Analysis.

**Methods s2** Hierarchical clustering analysis.

**Results s1** Hierarchical clustering analysis.

**Figure s1** PRISMA flowchart for data selection in coordinate-based meta-analysis.

**Figure s2** PRISMA checklist.

**Figure s3** Dendrogram of the hierarchical clustering and heatmap of the distance-dissimilarity.

**Figure s4** Dendrogram of the hierarchical clustering visualized according to the CR proxy.

**Figure s5** Dendrogram of the hierarchical clustering visualized according to the task type.

**Table s1** Details of the included studies about CR proxies, fMRI tasks, sample size, brain coordinates of activation foci, t value and type of contrast.

**Table s2** Output of the Jackknife (leave-one-out) analysis.

**References.**

This supplementary material has been provided by the authors to give readers additional information about their work.

**Methods s1 Creation of experiment level maps for Unsupervised Machine Learning Analysis**

Since high heterogeneity was expected in tb-fMRI in light of the variety of study designs, cognitive tasks, and CR proxy definitions, complementary machine learning analysis were implemented. In fact, while CBMA explicitly tests the consistency among experiments, machine learning allows us to explore the existence of hidden patterns or regularities through a data-mining and hypothesis-free approach. The input for both hierarchical clustering and one-class SVM analyses (Methods s1) consisted of a set of modelled activation (MA) maps, one for each of the 12 tb-fMRI experiments considered. A MA map represents the estimate of the original whole brain results obtained at the end of an experiment, based on the coordinates of the foci made available in the published article. The maps were spatially standardized and smoothed using kernels that account for sample size, thereby modeling spatial uncertainty in a data-driven manner. This procedure ensured that all experiments were represented in a common anatomical space with standardized spatial resolution before performing machine learning analyses (Eickhoff et al., 2009; Yang et al., 2016).

**Methods s2 Hierarchical clustering analysis**

Hierarchical clustering was conducted to identify potential subgroups within the dataset based on voxel-wise similarity of MA maps. As a data-driven unsupervised learning technique, hierarchical clustering is extensively applied in MRI research to reveal nested patterns of similarity across subjects or experiments (Khosla et al., 2019; Manuello, Mancuso, et al., 2022; Manuello, Verdejo-Román, et al., 2022; Mirzaei & Adeli, 2018). Unlike partition-based algorithms such as k-means, hierarchical clustering does not require the a priori specification of the number of clusters (Khosla et al., 2019). Instead, it iteratively organizes data into a tree-like structure of nested partitions, allowing for a flexible exploration of the underlying structure of the dataset (Khosla et al., 2019). Similarity between activation patterns was computed using the inverse correlation metric (1−r), where r represents the Pearson correlation coefficient between the MA maps of two experiments. Clustering was performed using Ward’s linkage method (Ward, 1963), which minimizes total within-cluster variance at each step, thus ensuring internal cluster homogeneity (Khosla et al., 2019). To visualize the results, a dendrogram was generated to illustrate the hierarchical structure and dissimilarity levels at which clusters merge. A heatmap of the dissimilarity matrix was also produced to further explore pairwise relationships between experiments**.**

**Results s1 Hierarchical clustering analysis**

Hierarchical clustering analysis did not yield distinct subgroups within the dataset, as most experiments clustered at high dissimilarity levels (i.e. very close to the top branch of the dendrogram). While some clusters emerged, their separation on the distance value was minimal, suggesting that the underlying structure lacked clear differentiation. Specifically, a cluster comprising three experiments: CR-related negative contrast in (Rodríguez-Aranda et al., 2020), CR-related positive contrast in (Stern et al., 2018), and CR-related negative contrast in (Bartrés-Faz et al., 2009), emerged at a lower level of dissimilarity compared to the main cluster of the remaining studies. Despite this localized clustering, overall dissimilarity across the dataset remained high, reflecting substantial heterogeneity in activation patterns. The dissimilarity matrix, visualized as a heatmap, illustrated consistently high dissimilarity values across most experiment pairs, with limited pockets of local similarity (Figure s3). To investigate potential factors influencing the clustering patterns, experiments were color-coded in the dendrograms according to their respective CR proxy categories (education, IQ, or composite score; Figure s4) and the cognitive domain of the experimental task (memory, semantic verbal fluency, visual processing/encoding, or multi-domain cognitive tasks; Figure s5).

**Fig. s1** PRISMA flowchart for data selection in coordinate-based meta-analysis.


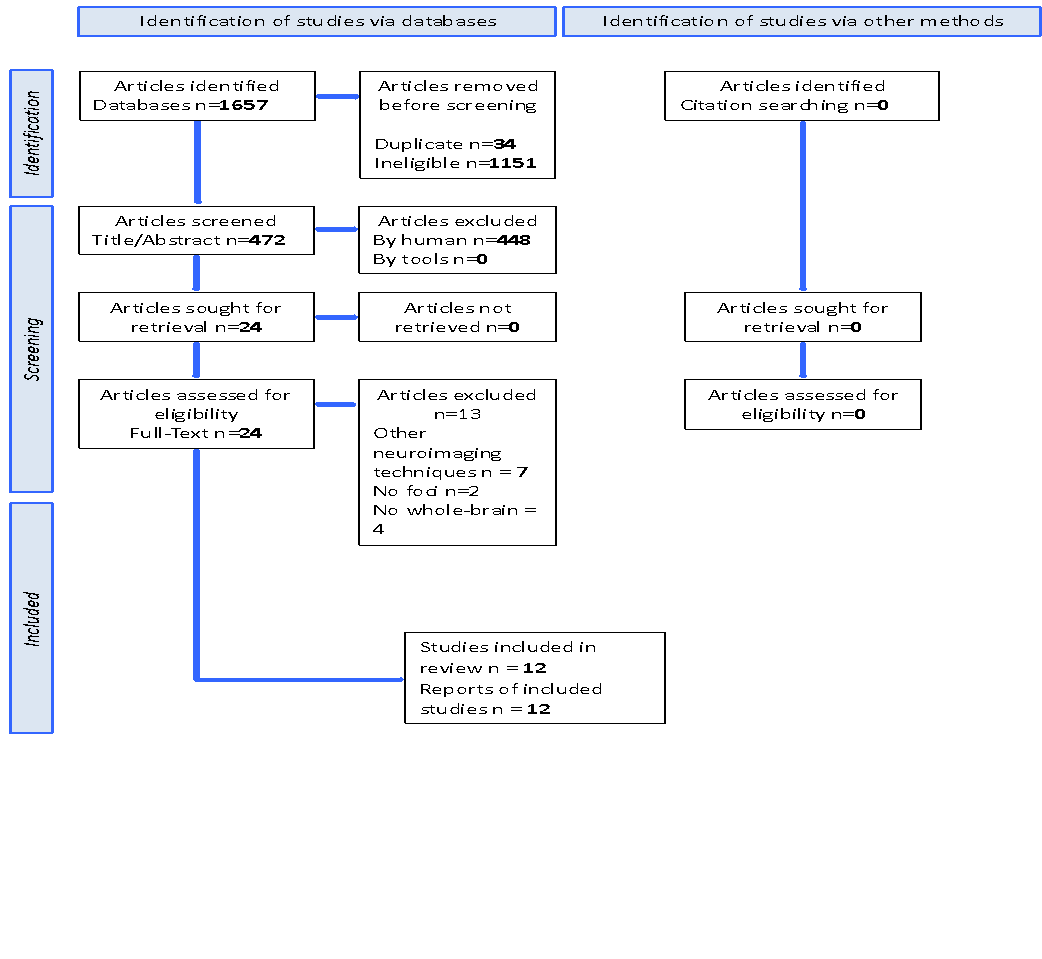


**Fig. s2** PRISMA checklist.

| **Section and Topic** | **Item #** | **Checklist item** | **Location where item is reported** |
| --- | --- | --- | --- |
| **TITLE** | | |  |
| Title | 1 | Identify the report as a systematic review. | Title |
| **ABSTRACT** | | |  |
| Abstract | 2 | See the PRISMA 2020 for Abstracts checklist. | Abstract |
| **INTRODUCTION** | | |  |
| Rationale | 3 | Describe the rationale for the review in the context of existing knowledge. | 1. Introduction |
| Objectives | 4 | Provide an explicit statement of the objective(s) or question(s) the review addresses. | 1. Introduction |
| **METHODS** | | |  |
| Eligibility criteria | 5 | Specify the inclusion and exclusion criteria for the review and how studies were grouped for the syntheses. | 2.2 Eligibility criteria, study selection and data extraction |
| Information sources | 6 | Specify all databases, registers, websites, organisations, reference lists and other sources searched or consulted to identify studies. Specify the date when each source was last searched or consulted. | 2.1 Literature search strategy |
| Search strategy | 7 | Present the full search strategies for all databases, registers and websites, including any filters and limits used. | 2.1 Literature search strategy |
| Selection process | 8 | Specify the methods used to decide whether a study met the inclusion criteria of the review, including how many reviewers screened each record and each report retrieved, whether they worked independently, and if applicable, details of automation tools used in the process. | 2.2 Eligibility criteria, study selection and data extraction |
| Data collection process | 9 | Specify the methods used to collect data from reports, including how many reviewers collected data from each report, whether they worked independently, any processes for obtaining or confirming data from study investigators, and if applicable, details of automation tools used in the process. | 2.2 Eligibility criteria, study selection and data extraction |
| Data items | 10a | List and define all outcomes for which data were sought. Specify whether all results that were compatible with each outcome domain in each study were sought (e.g. for all measures, time points, analyses), and if not, the methods used to decide which results to collect. | Figure s1 – Table 2 |
|  | 10b | List and define all other variables for which data were sought (e.g. participant and intervention characteristics, funding sources). Describe any assumptions made about any missing or unclear information. | Table 1 – Table 2 |
| Study risk of bias assessment | 11 | Specify the methods used to assess risk of bias in the included studies, including details of the tool(s) used, how many reviewers assessed each study and whether they worked independently, and if applicable, details of automation tools used in the process. | 2.2 Eligibility criteria, study selection and data extraction |
| Effect measures | 12 | Specify for each outcome the effect measure(s) (e.g. risk ratio, mean difference) used in the synthesis or presentation of results. | 2.2 Eligibility criteria, study selection and data extraction |
| Synthesis methods | 13a | Describe the processes used to decide which studies were eligible for each synthesis (e.g. tabulating the study intervention characteristics and comparing against the planned groups for each synthesis (item #5)). | Table 2 |
|  | 13b | Describe any methods required to prepare the data for presentation or synthesis, such as handling of missing summary statistics, or data conversions. | p.4 - Figure s1 |
|  | 13c | Describe any methods used to tabulate or visually display results of individual studies and syntheses. | n/a |
|  | 13d | Describe any methods used to synthesize results and provide a rationale for the choice(s). If meta-analysis was performed, describe the model(s), method(s) to identify the presence and extent of statistical heterogeneity, and software package(s) used. | 2.3.1 CBMA with the PSI-SDM algorithm; 2.3.2 Reliability and Heterogeneity analyses |
|  | 13e | Describe any methods used to explore possible causes of heterogeneity among study results (e.g. subgroup analysis, meta-regression). | 2.3.2 Reliability and Heterogeneity analyses; 2.3.3 Meta-regression analysis with PSI-SDM |
|  | 13f | Describe any sensitivity analyses conducted to assess robustness of the synthesized results. | 2.3.2 Reliability and Heterogeneity analyses; |
| Reporting bias assessment | 14 | Describe any methods used to assess risk of bias due to missing results in a synthesis (arising from reporting biases). | n/a |
| Certainty assessment | 15 | Describe any methods used to assess certainty (or confidence) in the body of evidence for an outcome. | 2.3.2 Reliability and Heterogeneity analyses; |
| **RESULTS** | | |  |
| Study selection | 16a | Describe the results of the search and selection process, from the number of records identified in the search to the number of studies included in the review, ideally using a flow diagram. | 3. Results |
|  | 16b | Cite studies that might appear to meet the inclusion criteria, but which were excluded, and explain why they were excluded. | 2.2 Eligibility criteria, study selection and data extraction |
| Study characteristics | 17 | Cite each included study and present its characteristics. | Table 2 – Table s1 |
| Risk of bias in studies | 18 | Present assessments of risk of bias for each included study. | n/a |
| Results of individual studies | 19 | For all outcomes, present, for each study: (a) summary statistics for each group (where appropriate) and (b) an effect estimate and its precision (e.g. confidence/credible interval), ideally using structured tables or plots. | Table 2 – Figure 2 – Table s1 |
| Results of syntheses | 20a | For each synthesis, briefly summarise the characteristics and risk of bias among contributing studies. | 3.1 Findings from the literature; 3.1.2 fMRI task paradigms of studies included |
|  | 20b | Present results of all statistical syntheses conducted. If meta-analysis was done, present for each the summary estimate and its precision (e.g. confidence/credible interval) and measures of statistical heterogeneity. If comparing groups, describe the direction of the effect. | 3.2.1 PSI-SDM Meta-analysis |
|  | 20c | Present results of all investigations of possible causes of heterogeneity among study results. | 3.2.2 Reliability and Heterogeneity analyses; 3.2.3 Meta-regression analysis with PSI-SDM |
|  | 20d | Present results of all sensitivity analyses conducted to assess the robustness of the synthesized results. | 3.2.2 Reliability and Heterogeneity analyses |
| Reporting biases | 21 | Present assessments of risk of bias due to missing results (arising from reporting biases) for each synthesis assessed. | n/a |
| Certainty of evidence | 22 | Present assessments of certainty (or confidence) in the body of evidence for each outcome assessed. | 3.2.2 Reliability and Heterogeneity analyses |
| **DISCUSSION** | | |  |
| Discussion | 23a | Provide a general interpretation of the results in the context of other evidence. | 4. Discussion |
|  | 23b | Discuss any limitations of the evidence included in the review. | 4.1 Open issues in the study of CR |
|  | 23c | Discuss any limitations of the review processes used. | 4.2 Methodological Considerations |
|  | 23d | Discuss implications of the results for practice, policy, and future research. | 4.3 Towards a network-based approach in CR research; 5. Conclusion |
| **OTHER INFORMATION** | | |  |
| Registration and protocol | 24a | Provide registration information for the review, including register name and registration number, or state that the review was not registered. | n/a |
|  | 24b | Indicate where the review protocol can be accessed, or state that a protocol was not prepared. | n/a |
|  | 24c | Describe and explain any amendments to information provided at registration or in the protocol. | n/a |
| Support | 25 | Describe sources of financial or non-financial support for the review, and the role of the funders or sponsors in the review. | Acknowledgements |
| Competing interests | 26 | Declare any competing interests of review authors. | Competing Interests |
| Availability of data, code and other materials | 27 | Report which of the following are publicly available and where they can be found: template data collection forms; data extracted from included studies; data used for all analyses; analytic code; any other materials used in the review. | Supplementary materials |

**Fig. s3** Dendrogram of the hierarchical clustering and heatmap of the distance-dissimilarity.

*
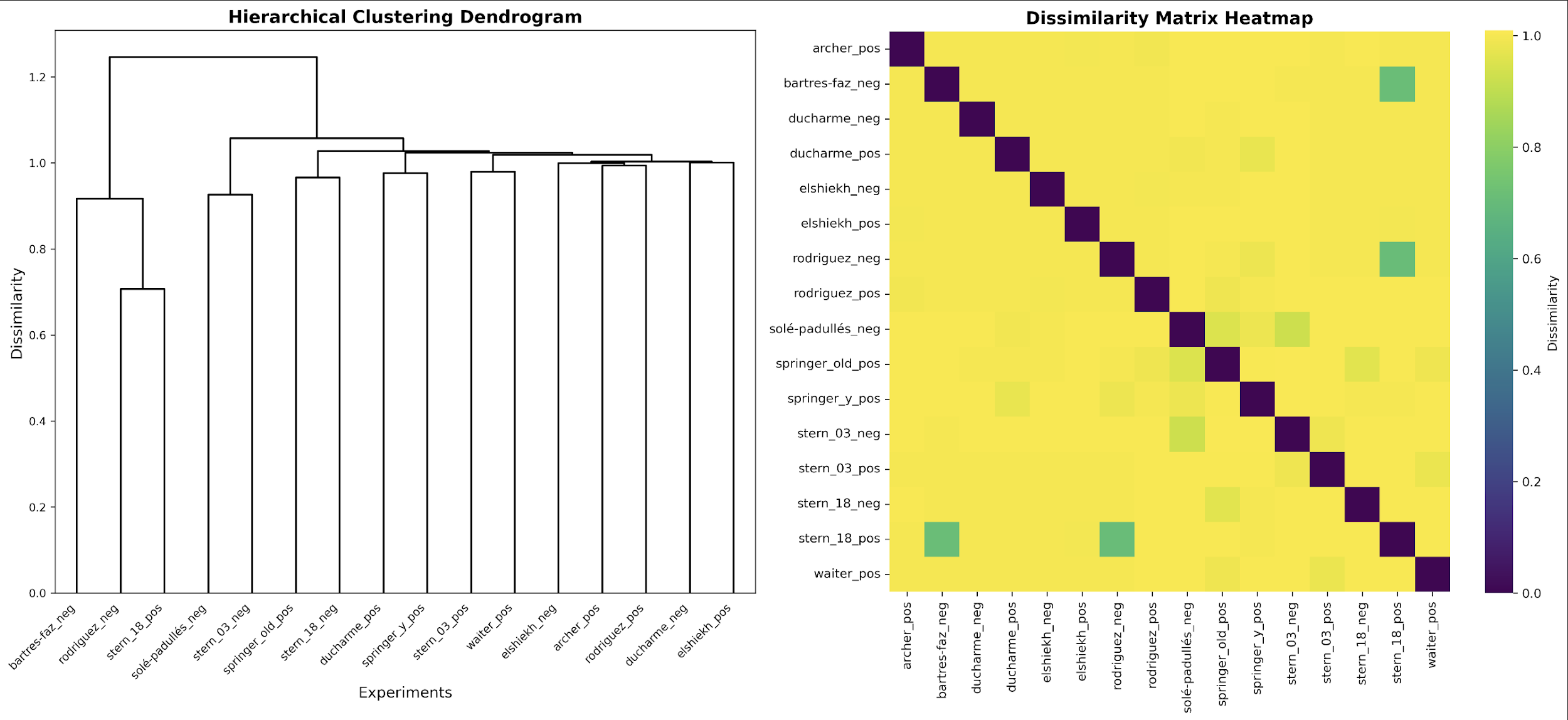
*

*Abbreviations: MAs, modelled activation maps*.

**Fig. s4** Dendrogram of the hierarchical clustering visualized according to the CR proxy.

***
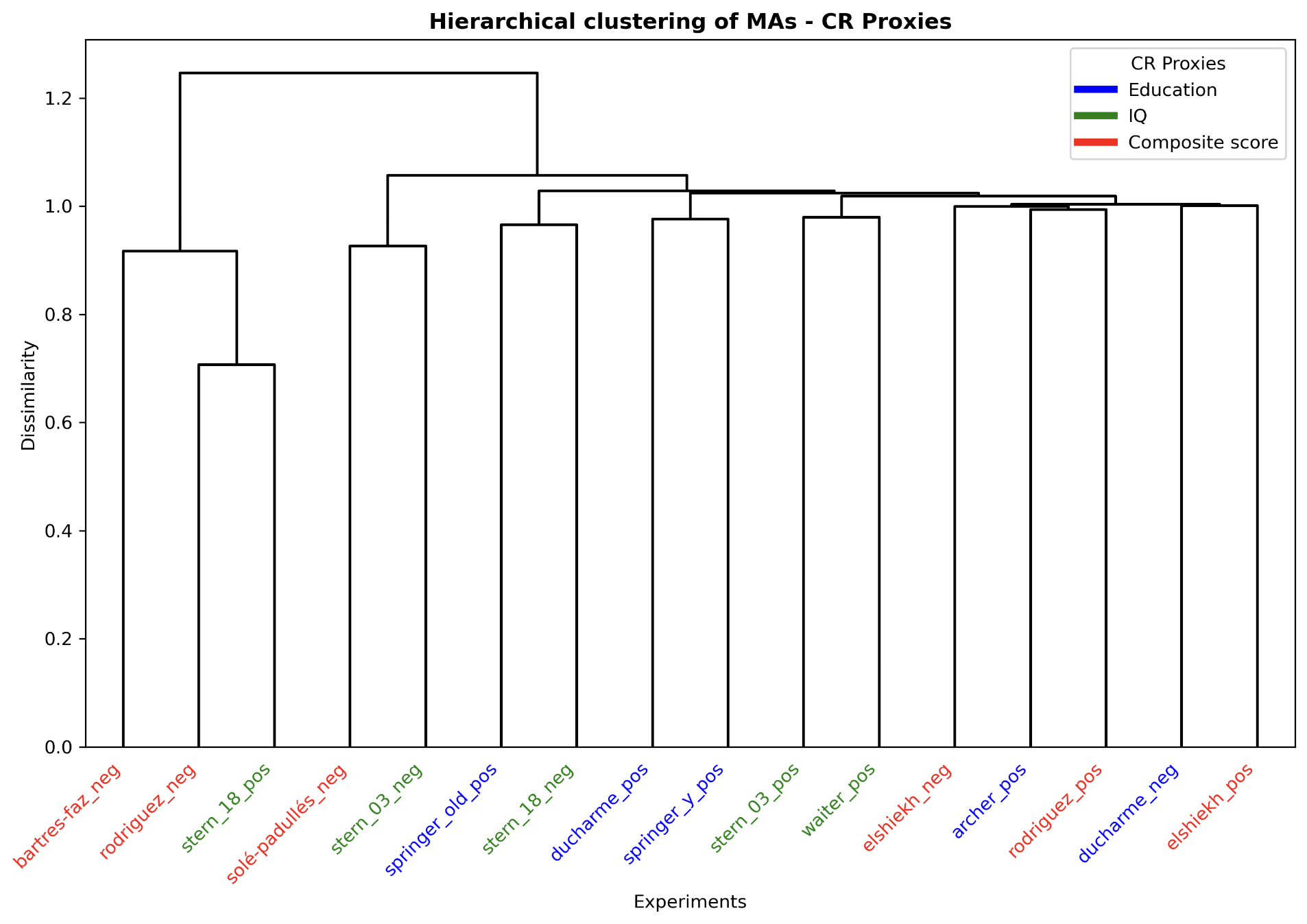
****Abbreviations: MAs, modelled activation maps*.**Fig. s5**Dendrogram of the hierarchical clustering visualized according to the task type.

***
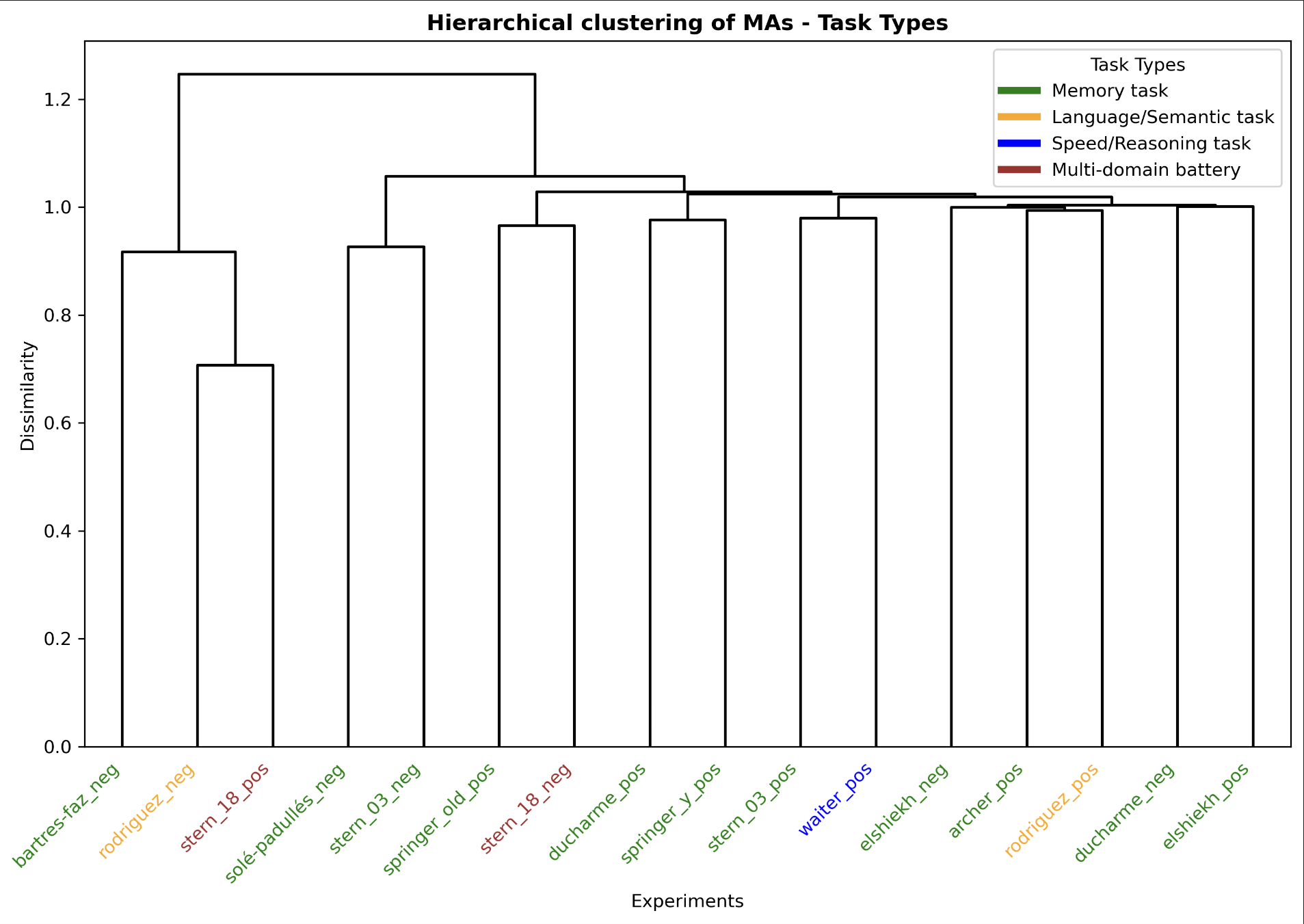
****Abbreviations: MAs, modelled activation maps*.

**Table s1** Details of the included studies about CR proxies, fMRI tasks, sample size, brain coordinates of activation foci, t value and type of contrast.

| **Author, Year** | **DOI** | **CR Proxy** | **fMRI Task Domain** | **N** | **x** | **y** | **z** | **t-value** | **CR - tb-fMRI positive association** | **CR - tb-fMRI negative association** |
| --- | --- | --- | --- | --- | --- | --- | --- | --- | --- | --- |
| Archer et al., (2018) | 10.1371/journal.pone.0194878 | Education | Working Memory | 189 | -48.68 | -0.19 | -7.64 | 4.20 | 1 | 0 |
|  |  |  |  |  | 15.42 | -76.32 | 16.8 | 4.49 | 1 | 0 |
|  |  |  |  |  | -9.61 | -62.91 | -6.79 | 4.39 | 1 | 0 |
|  |  |  |  |  | -51.48 | -0.17 | -7.67 | 3.82 | 1 | 0 |
| Bartrés-Faz et al., (2009) | 10.1016/j.biopsycho.2008.10.005 | Composite score | Working Memory | 15 | 51,00 | -13,00 | 14,00 | -7.9 | 0 | 1 |
| Ducharme-Laliberté et al., (2022) | 10.1016/j.neurobiolaging.2022.02.001 | Education | Working Memory | 39 | -9.7 | 36.38 | 51.7 | 5.87 | 0 | 1 |
|  |  |  |  |  | 12.79 | -15.38 | 23.45 | 5.32 | 1 | 0 |
| Elshiekh et al., (2020) | 10.1016/j.cortex.2020.05.003 | Composite score | Episodic Memory | 154 | -46 | 8 | 26 | -3.84 | 0 | 1 |
|  |  |  |  |  | -53 | -16 | -5 | 4.53 | 1 | 0 |
|  |  |  |  |  | 9 | -94 | 24 | 5.58 | 1 | 0 |
| Rodríguez-Aranda et al., (2020)*young | 10.3389/fnhum.2020.00203 | Composite Score | Language/Semantic memory | 15 | 8.13 | -47 | 5.52 | -4.3 | 0 | 1 |
| Rodríguez-Aranda et al., (2020)*old | 10.3389/fnhum.2020.00203 | Composite Score | Language/Semantic memory | 27 | -44.27 | 18.84 | 37.26 | 4.8 | 1 | 0 |
|  |  |  |  |  | 21.07 | 13.18 | 55.61 | 5.6 | 1 | 0 |
|  |  |  |  |  | -40.8 | -82.23 | 23.8 | 4.7 | 1 | 0 |
|  |  |  |  |  | 11.6 | -71.6 | 28.87 | 4.7 | 1 | 0 |
| Solé-Padullés et al., (2009) | 10.1016/j.neurobiolaging.2007.10.008 | Composite Score | Episodic memory | 16 | -36 | -9 | 24 | -9.62 | 0 | 1 |
|  |  |  |  |  | -7 | -38 | -37 | -9.3 | 0 | 1 |
|  |  |  |  |  | -18 | -25 | 5 | -8.13 | 0 | 1 |
|  |  |  |  |  | 43 | -48 | -4 | -8.04 | 0 | 1 |
|  |  |  |  |  | 8 | -40 | -36 | -7.76 | 0 | 1 |
|  |  |  |  |  | 8 | -15 | 48 | -7.09 | 0 | 1 |
|  |  |  |  |  | -4 | -17 | 38 | -5.9 | 0 | 1 |
|  |  |  |  |  | 24 | -17 | 15 | -5.74 | 0 | 1 |
|  |  |  |  |  | 33 | -7 | 26 | -7.74 | 0 | 1 |
|  |  |  |  |  | -2 | -46 | -28 | -7.09 | 0 | 1 |
| Springer et al., (2005)*young | 10.1037/0894-4105.19.2.181 | Education | Episodic memory | 14 | 2.12 | -36.42 | 62.26 | 6.1 | 1 | 0 |
|  |  |  |  |  | -5.37 | -47.36 | 57.69 | 7.7 | 1 | 0 |
|  |  |  |  |  | 6.15 | -22.56 | 30.92 | 11.1 | 1 | 0 |
|  |  |  |  |  | -12.55 | -18.67 | 31.02 | 7.6 | 1 | 0 |
|  |  |  |  |  | 17.75 | -22.62 | -15.89 | 3.6 | 1 | 0 |
|  |  |  |  |  | 24.86 | -62.87 | 13.47 | 4.6 | 1 | 0 |
|  |  |  |  |  | -27.48 | -32.73 | 18.89 | 4.3 | 1 | 0 |
|  |  |  |  |  | 20.89 | -35.97 | 55.29 | 6.8 | 1 | 0 |
|  |  |  |  |  | 43.45 | -56.94 | 32.21 | 7.3 | 1 | 0 |
|  |  |  |  |  | -16.56 | -43.23 | 54.28 | 7.3 | 1 | 0 |
|  |  |  |  |  | 20.9 | -68.64 | 38.24 | 5.3 | 1 | 0 |
|  |  |  |  |  | 13.7 | -58.45 | 6.47 | 4.6 | 1 | 0 |
|  |  |  |  |  | -12.61 | -84.87 | 7.67 | 4.4 | 1 | 0 |
|  |  |  |  |  | 32.19 | -75.36 | 27 | 7.6 | 1 | 0 |
|  |  |  |  |  | 51.26 | -61.02 | -11.35 | 4.2 | 1 | 0 |
|  |  |  |  |  | 13.81 | -20.87 | 9.48 | 8.2 | 1 | 0 |
|  |  |  |  |  | 2.68 | -27.15 | -5.59 | 6.6 | 1 | 0 |
| Springer et al., (2005)*old | 10.1037/0894-4105.19.2.181 | Education | Episodic memory | 19 | 28.72 | 11.11 | 33.87 | 3.3 | 1 | 0 |
|  |  |  |  |  | -49.81 | 26.98 | 30.63 | 4 | 1 | 0 |
|  |  |  |  |  | -23.93 | 5.39 | 61.69 | 4.6 | 1 | 0 |
|  |  |  |  |  | 17.81 | 25.37 | -1.21 | 4.2 | 1 | 0 |
|  |  |  |  |  | 47.24 | -41.93 | 33.46 | 3.1 | 1 | 0 |
|  |  |  |  |  | 47.54 | -42.49 | -6.3 | 4.1 | 1 | 0 |
|  |  |  |  |  | 58.93 | -19.15 | -15.13 | 3.7 | 1 | 0 |
| Stern et al., (2003) | 10.1076/jcen.25.5.691.14573 | IQ | Episodic memory | 19 | 8 | 58 | 36 | -8.34 | 0 | 1 |
|  |  |  |  |  | 20 | 56 | 3 | -6.37 | 0 | 1 |
|  |  |  |  |  | 16 | 66 | 2 | -5.47 | 0 | 1 |
|  |  |  |  |  | 30 | 47 | 1 | -5.8 | 0 | 1 |
|  |  |  |  |  | -26 | 43 | 3 | -5.78 | 0 | 1 |
|  |  |  |  |  | 55 | -10 | 30 | -5.69 | 0 | 1 |
|  |  |  |  |  | 40 | -25 | 1 | -5.57 | 0 | 1 |
|  |  |  |  |  | 24 | 47 | 12 | -5.09 | 0 | 1 |
|  |  |  |  |  | -6 | -39 | -37 | -4.99 | 0 | 1 |
|  |  |  |  |  | 8 | -50 | -34 | -4.71 | 0 | 1 |
|  |  |  |  |  | -55 | -35 | 4 | -6.68 | 0 | 1 |
|  |  |  |  |  | -46 | -33 | 9 | -5.07 | 0 | 1 |
|  |  |  |  |  | 16 | 68 | 2 | -6.14 | 0 | 1 |
|  |  |  |  |  | 24 | 49 | 10 | -6 | 0 | 1 |
|  |  |  |  |  | -16 | 49 | 9 | -5.84 | 0 | 1 |
|  |  |  |  |  | 57 | 4 | 33 | -5.58 | 0 | 1 |
|  |  |  |  |  | -30 | -60 | 14 | -5.5 | 0 | 1 |
|  |  |  |  |  | -24 | 42 | -16 | -5.5 | 0 | 1 |
|  |  |  |  |  | 63 | -34 | 24 | -5.43 | 0 | 1 |
|  |  |  |  |  | -36 | -34 | 64 | -5.43 | 0 | 1 |
|  |  |  |  |  | -26 | -12 | 67 | -5.32 | 0 | 1 |
|  |  |  |  |  | -18 | -35 | 9 | -5.32 | 0 | 1 |
|  |  |  |  |  | -22 | -55 | 65 | -5.22 | 0 | 1 |
|  |  |  |  |  | 55 | -36 | 15 | -5.13 | 0 | 1 |
|  |  |  |  |  | 42 | -42 | 61 | -5.08 | 0 | 1 |
|  |  |  |  |  | -63 | -27 | 40 | -5.05 | 0 | 1 |
|  |  |  |  |  | -48 | -44 | 19 | -4.91 | 0 | 1 |
|  |  |  |  |  | 30 | -10 | 65 | -4.89 | 0 | 1 |
|  |  |  |  |  | -14 | -14 | -11 | -4.75 | 0 | 1 |
|  |  |  |  |  | 32 | -12 | 63 | -4.62 | 0 | 1 |
|  |  |  |  |  | 38 | -25 | -2 | -4.59 | 0 | 1 |
|  |  |  |  |  | 55 | -19 | 38 | -4.56 | 0 | 1 |
|  |  |  |  |  | 22 | -26 | 71 | -4.56 | 0 | 1 |
|  |  |  |  |  | -20 | 54 | -11 | 7.06 | 1 | 0 |
|  |  |  |  |  | -48 | -15 | 54 | 5.79 | 1 | 0 |
|  |  |  |  |  | 16 | 48 | -6 | 5.29 | 1 | 0 |
| Stern et al., (2018) | 10.1016/j.neuroimage.2018.05.033 | IQ | Vocabulary,  Perceptual Speed,  Fluid Reasoning,  Episodic Memory | 255 | 32.2 | -49.78 | 38.07 | -3.3 | 0 | 1 |
|  |  |  |  |  | 35.2 | 6.8 | 39.92 | -2.9 | 0 | 1 |
|  |  |  |  |  | -35.18 | -52.13 | 37.1 | -3.01 | 0 | 1 |
|  |  |  |  |  | -37.44 | 43.52 | 12.24 | -2.96 | 0 | 1 |
|  |  |  |  |  | 4.4 | -49.57 | 2.56 | 3.3 | 1 | 0 |
|  |  |  |  |  | -1.07 | 31.12 | 22.5 | 2.99 | 1 | 0 |
|  |  |  |  |  | -57.25 | -9.9 | 7.73 | 2.58 | 1 | 0 |
|  |  |  |  |  | 49.4 | -8.04 | 11.82 | 2.73 | 1 | 0 |
| Waiter et al., (2008) | 10.1016/j.neuroimage.2008.02.045 | IQ | Processing speed | 40 | 4 | 41 | -5 | 5.81 | 1 | 0 |
|  |  |  |  |  | 2 | 47 | 5 | 4.4 | 1 | 0 |
|  |  |  |  |  | 10 | 35 | 0 | 3.41 | 1 | 0 |

*Abbreviations: CR, Cognitive Reserve; IQ, Intelligent Quotient.*

**Table s2** Output of the Jackknife (leave-one-out) analysis.

| **AUTHOR, YEAR OF THE**  **LEFT-OUT EXPERIMENT** | **Clusters found** |
| --- | --- |
|  |  |
| Archer et al. (2018) | No |
| Bartres-Faz et al. (2009) | No |
| Ducharme-Laliberté et al. (2022) | No |
| Elshiekh et al. (2020) | No |
| Rodríguez-Aranda et al. (2020) *young | No |
| Rodríguez-Aranda et al. (2020) *old | No |
| Sole-padulles et al. (2009) | No |
| Springer et al. (2005) *young | No |
| Springer et al. (2005) *old | No |
| Stern et al. (2003) | No |
| Stern et al. (2018) | No |
| Waiter et al. (2008) | No |

**References**

Bartrés-Faz D, Solé-Padullés C, Junqué C, et al. (2009) Interactions of cognitive reserve with regional brain anatomy and brain function during a working memory task in healthy elders. Biol Psychol 80(2):256–259.

Pimentel MAF, Clifton DA, Clifton L, et al. (2014) A review of novelty detection. Signal Process 99:215–249.

Rodríguez-Aranda C, Castro-Chavira SA, Espenes R, et al. (2020) The Role of Moderating Variables on BOLD fMRI Response During Semantic Verbal Fluency and Finger Tapping in Active and Educated Healthy Seniors. Front Hum Neurosci 14:203.

Schölkopf B, Platt JC, Shawe-Taylor J, et al. (2001) Estimating the Support of a High-Dimensional Distribution. Neural Comput 13(7):1443–1471.

Springer MV, McIntosh AR, Winocur G, et al. (2005) The Relation Between Brain Activity During Memory Tasks and Years of Education in Young and Older Adults. Neuropsychology 19(2):181–192.

Stern Y, Gazes Y, Razlighi Q, et al. (2018) A task-invariant cognitive reserve network. NeuroImage 178:36–45.

Stern Y, Zarahn E, Hilton HJ, et al. (2003) Exploring the Neural Basis of Cognitive Reserve. J Clin Exp Neuropsychol25(5):691–701.
